# Changes in Eating Behavior and Food Preferences After Semaglutide or Tirzepatide Initiation: A Prospective Observational Pilot Study

**DOI:** 10.3390/nu18213675

**Published:** 2026-11-05

**Authors:** Elisabetta Camajani, Francesco Frigerio, Chiara Quattrini, Valentina Franzese, Andrea Armani, Stefania Gorini, Tiziana Filardi, Alessandra Feraco, Massimiliano Caprio, Mauro Lombardo

**Affiliations:** 1 Department of Human Sciences and Promotion of the Quality of Life, San Raffaele Open University, Via di Val Cannuta, 247, 00166 Rome, Italy; elisabetta.camajani@uniroma5.it (E.C.); chiara.quattrini@uniroma5.it (C.Q.); valentina.franzese@unicampus.it (V.F.); andrea.armani@uniroma5.it (A.A.); stefania.gorini@uniroma5.it (S.G.); tiziana.filardi@uniroma5.it (T.F.); massimiliano.caprio@uniroma5.it (M.C.); mauro.lombardo@uniroma5.it (M.L.); 2 Laboratory of Cardiovascular Endocrinology, IRCCS San Raffaele Roma, Via di Val Cannuta, 247, 00166 Rome, Italy; 3 Campus Bio-Medico University of Rome, 00128 Rome, Italy; 4 Biochemical Pharmacology, William Harvey Research Institute, Queen Mary University of London, London E1 4NS, UK

**Keywords:** semaglutide, tirzepatide, obesity, eating behavior, food cravings, appetite regulation, incretin therapy

## Abstract

Background: This pilot study evaluated changes in eating behavior, appetite, and dietary habits during semaglutide or tirzepatide treatment in adults with obesity. Methods: In this prospective observational study, 38 non-diabetic adults with obesity (mean age 56.1 ± 11.3 years; 73.7% female; BMI 32.7 ± 4.5 kg/m^2^) initiating semaglutide (n = 21) or tirzepatide (n = 17) were assessed at baseline and after 3 months. Clinical parameters and self-reported eating behaviors were evaluated using standardized measurements and a study-specific questionnaire. Results: Mean body weight decreased by 7.2 ± 4.9 kg (*p* < 0.001), and systolic blood pressure decreased by 6.1 ± 6.3 mm Hg (*p* < 0.001). Cravings for sweet and savory foods, difficulty resisting hunger between meals, and daily meal frequency decreased, whereas perceived meal-induced satiety increased (all *p* ≤ 0.005). A substantial reduction in meal portion size was reported by 84.2% of participants. Reported food-group consumption changed, but improved overall dietary quality was not established. Conclusions: Over 3 months, incretin-based treatment was accompanied by weight loss and changes in self-reported eating behavior. The observational design, absence of a control group, and unvalidated questionnaire limit interpretation. Larger controlled studies using validated measures are warranted.

## 1. Introduction

Obesity is a chronic, complex, and multifactorial disease characterized by excessive or abnormal adiposity that may impair health, and is associated with increased morbidity, mortality, and healthcare costs worldwide [1]. Beyond excess adiposity, obesity is characterized by complex alterations in appetite regulation, energy homeostasis, food reward processing, and eating behavior, which collectively contribute to the development and persistence of weight gain [2]. As a result, successful obesity management requires not only the achievement of weight loss, but also the modification of the behavioral and physiological drivers of excessive food intake.

Glucagon-like peptide-1 receptor agonists (GLP-1RAs) were initially developed for glycemic management in type 2 diabetes; weight reduction, first observed as an additional clinical effect of these agents, subsequently became a therapeutic target in its own right [3,4]. Semaglutide, a long-acting GLP-1 receptor agonist, and tirzepatide, a dual GIP/GLP-1 receptor agonist, have demonstrated efficacy in reducing body weight and improving metabolic outcomes in relevant clinical populations [5]. GLP-1 and GIP are endogenous incretin hormones involved in glucose-dependent insulin secretion. GLP-1 also suppresses glucagon secretion and delays gastric emptying, while GLP-1 receptor signaling contributes to appetite regulation and satiety. The effects of GLP-1 receptor agonists on appetite and food intake may involve central pathways associated with satiety and food reward, in addition to peripheral physiological effects [6,7,8]. GIP, in addition to its insulinotropic action, contributes to postprandial lipid handling in adipose tissue and has glucose-dependent effects on glucagon secretion [9]; GIP receptors are also expressed in brain areas involved in the control of food intake and nausea, and it has been proposed that GIP receptor agonism may contribute to reduced food intake and attenuate GLP-1RA-related nausea [10]. These mechanisms provide a rationale for investigating changes in eating behavior during treatment, but they do not establish the mechanism underlying any behavioral changes observed in an individual observational cohort.

Although the anthropometric efficacy of these agents is well established, less is known about their specific impact on patients’ everyday eating behaviors, taste perceptions, and dietary choices in routine clinical practice. Emerging evidence suggests that incretin-based therapies may alter food preferences, suppress food cravings, improve perceived control over eating, and affect the desirability of highly palatable foods [11]. However, changes in self-reported appetite and food preferences should be distinguished from objectively measured food intake and from demonstrated neurobiological mechanisms. Identifying early changes in hunger control, sweet and savory cravings, meal-induced satiety, and food selection patterns may help characterize the behavioral changes accompanying pharmacological weight management.

Therefore, this prospective observational pilot study aimed to evaluate changes in eating behavior, appetite perception, and dietary habits in adults with obesity treated with semaglutide or tirzepatide over a 3-month period. Using a structured self-administered questionnaire, we investigated changes in food cravings, difficulty resisting hunger between meals, perceived satiety, daily meal frequency, and reported consumption of specific food categories. We also assessed changes in body weight and blood pressure and examined associations between behavioral changes, gastrointestinal symptoms, and clinical response. The study was exploratory and was not designed to establish causal effects or identify the biological mechanisms mediating weight loss.

## 2. Materials and Methods

### 2.1. Study Population and Design

This prospective, observational pilot study assessed changes in eating behavior, appetite perception, and dietary habits among adult patients with obesity who initiated treatment with semaglutide or tirzepatide within routine clinical practice. The study protocol was approved by the local Ethics Committee (Approval date: 05-11-2025 - GLP1-TASTE 422/SR/25—CET Lazio Area 5) and registered on ClinicalTrials.gov (NCT07229170 Approval date: 10-11-2025). All participants provided written informed consent prior to enrollment, in accordance with the ethical principles of the Declaration of Helsinki.

Participants were assessed at baseline (T0), before the initiation of pharmacological treatment, and reassessed after 3 months of treatment (T1). The inclusion criteria were: adult patients (aged ≥18 years) with a diagnosis of obesity (BMI ≥ 30 kg/m^2^ or BMI ≥ 27 kg/m^2^ with weight-related comorbidities) initiating semaglutide or tirzepatide in routine outpatient clinical practice, who completed both baseline and 3-month follow-up evaluations. The exclusion criteria were: pregnancy or lactation, severe cognitive or psychiatric impairment hindering questionnaire completion, prior bariatric surgery, or active severe gastrointestinal disorders precluding incretin therapy. The decision to initiate treatment was made by the treating physician according to routine clinical evaluation, individual patient characteristics, and local prescribing guidelines. Previous exposure to GLP-1 receptor agonists or dual GIP/GLP-1 receptor agonists was defined as an exclusion criterion to ensure an incretin-naïve cohort at baseline. Baseline questionnaire completion and administration of the first treatment dose occurred on the same day during the initial clinical consultation.

Treatment was prescribed as part of routine clinical care rather than assigned by the study protocol. Dose titration was individualized according to treatment tolerability, gastrointestinal symptoms, and weight-loss response assessed during follow-up visits. Therefore, treatment selection was not randomized, and differences between treatment groups may reflect clinical prescribing decisions or baseline patient characteristics. The study did not include a control group. The flow of participants through enrollment, treatment initiation, follow-up, and final analysis is depicted in Supplementary Figure S1. Treatment adherence was monitored clinically during routine follow-up visits. However, quantitative adherence measures, such as pen counts or pharmacy refill records, were not systematically collected. Consequently, treatment adherence could not be quantified or incorporated into the analyses.

### 2.2. Clinical, Anthropometric, and Hemodynamic Assessments

At baseline and at the 3-month follow-up visit, clinical, anthropometric, and hemodynamic parameters were measured using standardized procedures. Body weight and height were assessed with participants wearing light clothing and no shoes, and body mass index (BMI) was calculated as weight in kilograms divided by the square of height in meters. Absolute body weight change was determined as follow-up weight minus baseline weight, and relative weight change was expressed as a percentage of baseline body weight. Systolic and diastolic blood pressures were recorded in duplicate after a 5-min rest in a seated position using a validated digital sphygmomanometer, and the two readings were averaged for analysis.

### 2.3. Assessment of Eating Behavior, Appetite, and Dietary Habits

At baseline and after 3 months of treatment, participants completed a structured, self-administered questionnaire designed to investigate eating behavior, appetite-related perceptions, and dietary habits. The questionnaire was adapted from tools previously applied by our research group in nutritional and behavioral studies [12] and further modified for the objectives of the present study. No formal psychometric validation was performed for this specific version of the questionnaire; therefore, its scores should be interpreted as exploratory self-reported measures rather than as validated clinical scales. The modifications introduced for the present study were the inclusion of specific items evaluating GLP-1RA-induced changes in sweet versus savory food cravings, difficulty resisting hunger between meals, meal-induced satiety, and gastrointestinal symptoms including nausea and taste alterations.

The baseline questionnaire assessed meal frequency, including snacks; timing of hunger throughout the day; meal skipping; eating speed; sleep-related and nocturnal eating behaviors; boredom-related and uncontrolled eating; sweet and savory food cravings; difficulty managing hunger between meals; and perceived meal-induced satiety. Dietary habits were assessed by recording the reported consumption of food categories, including dairy products, plant-based beverages, fresh cheeses, white meat, red meat, processed meats, fish, eggs, legumes, cooked and raw vegetables, fresh fruit, whole grains, nuts, soy-based products, and dark chocolate. Daily fluid intake was also recorded.

At the 3-month follow-up, participants completed the same core questionnaire to enable within-subject comparisons. Additional follow-up items assessed perceived changes in sweet versus savory taste preferences, meal portion size, food-related pleasure, nausea, and taste alterations. Behavioral items, craving scores, and gastrointestinal symptoms were rated using 10-point Likert scales, with higher scores indicating greater intensity of the assessed domain. The item-specific scoring rules and interpretation were as follows: continuous items were scored from 0 (minimum intensity/absence of perception) to 10 (maximum intensity/severe perception), with absolute changes calculated as follow-up score (T1) minus baseline score (T0). For qualitative shifts, categorical responses were recorded as presence (“YES”) or absence (“NO”) of regular consumption across food categories. All questionnaires were linked to clinical data using unique identification codes.

### 2.4. Statistical Analysis

Categorical variables were summarized as absolute frequencies and corresponding percentages, with proportions compared using Fisher’s exact test. Continuous variables were tested for normality using the Shapiro–Wilk test. Normally distributed variables were expressed as mean and standard deviation, and paired Student’s *t*-tests were used for within-subject comparisons between baseline and follow-up. Between-group comparisons of mean absolute changes between treatment arms were performed using Welch’s two-sample *t*-test. Bivariate correlation analyses were conducted using Pearson’s correlation coefficient to evaluate associations between changes in behavioral parameters, gastrointestinal symptom scores, and absolute weight loss. All statistical tests were two-tailed, and statistical significance was defined as a *p*-value less than 0.05. Analyses were performed using jamovi software (Version 2.7, The jamovi project, 2026).

## 3. Results

A total of 38 non-diabetic patients with obesity (mean age 56.1 ± 11.3 years; 73.7% female) were evaluated at baseline prior to the initiation of incretin-based therapy. Among them, 21 patients (55.3%) were prescribed Semaglutide and 17 patients (44.7%) received Tirzepatide. Dosing schedules followed a real-world titration protocol, starting at the lowest label-recommended dose (0.25 mg weekly for semaglutide and 2.5 mg weekly for tirzepatide) and progressively escalating based on individual gastrointestinal tolerability to achieve optimal weight loss outcomes. At the 3-month assessment, documented dose escalation followed label-recommended clinical pathways; individual maintenance dosages were tailored based on patient tolerability within routine clinical practice. The baseline demographic, anthropometric, hemodynamic, and dietary characteristics of the full cohort and subgroups stratified by gender and prescribed medication are detailed in Table 1. Overall, patients presented with a baseline body weight of 91.1 ± 14.5 kg and a BMI of 32.7 ± 4.5 kg/m^2^. When comparing treatment arms at baseline, patients on Tirzepatide presented with a significantly higher mean BMI relative to those on Semaglutide (35.4 ± 4.2 kg/m^2^ vs. 30.5 ± 3.4 kg/m^2^, *p* < 0.001). Because treatment allocation was not randomized, this baseline difference should be considered when interpreting comparisons between groups. The available data do not establish the reasons for the observed baseline difference.

Following 3 months of incretin-based therapy, significant improvements were observed in multiple clinical and behavioral parameters (Table 2). Information regarding the achieved dose at 3 months was available for 23 of 38 participants. Among semaglutide-treated patients, 13 of 16 subjects with available data had reached 1.0 mg weekly, whereas among tirzepatide-treated patients, the most commonly documented doses were 5.0 mg and 7.5 mg weekly (3 patients each). Because dose information was incomplete for a proportion of participants, dose–response analyses were not performed. Patients achieved a significant mean BMI reduction of −2.6 ± 1.6 kg/m^2^ (*p* < 0.001). Both systolic (137.4 ± 9.2 to 131.3 ± 10.3 mm Hg, *p* < 0.001) and diastolic blood pressure (85.6 ± 5.3 to 83.6 ± 6.7 mm Hg, *p* = 0.042) showed significant reductions. With respect to eating behavior, significant reductions were recorded in sweet craving (5.2 ± 3.1 vs. 3.3 ± 2.5, *p* < 0.001) and savory craving (6.9 ± 2.0 vs. 4.6 ± 2.4, *p* < 0.001). Patients reported reduced difficulty in resisting hunger (5.9 ± 2.7 vs. 3.1 ± 2.2, *p* < 0.001), increased meal-induced satiety (6.0 ± 2.3 vs. 7.5 ± 2.5, *p* = 0.003), and a lower daily meal frequency (4.3 ± 1.3 vs. 3.6 ± 1.0 meals/day, *p* = 0.005). Daily fluid intake did not change significantly (*p* = 0.201).

Subgroup analysis comparing 3-month absolute changes between patients treated with Semaglutide (*n* = 21) and Tirzepatide (*n* = 17) revealed comparable improvements across all clinical and behavioral parameters (Table 3). Both treatment arms achieved significant weight reduction, with a mean loss of −7.2 ± 4.5 kg and −7.3 ± 5.5 kg (*p* = 0.924). BMI reductions were −2.4 ± 1.4 kg/m^2^ in the Semaglutide group and −3.0 ± 1.9 kg/m^2^ in the Tirzepatide group (*p* = 0.258). Hemodynamic improvements were similarly balanced between groups, with systolic blood pressure reductions of −6.6 ± 5.9 mm Hg for Semaglutide vs. −5.5 ± 6.9 mm Hg for Tirzepatide (*p* = 0.589). Regarding eating behavior, both medications yielded marked reductions in food cravings and hunger perception. Craving for sweet foods decreased by −1.5 ± 2.6 with Semaglutide and −2.2 ± 2.9 with Tirzepatide (*p* = 0.435), whereas savory food craving declined by −2.7 ± 2.7 and −1.8 ± 2.6, respectively (*p* = 0.281). Both agents substantially reduced perceived difficulty in resisting hunger (−2.8 ± 3.0 vs. −2.9 ± 2.8, *p* = 0.849) and enhanced meal-induced satiety (+1.1 ± 2.7 vs. +2.1 ± 3.1, *p* = 0.324), with no statistically significant differences observed between the two incretin therapies.

Evaluation of self-reported qualitative dietary shifts at 3 months revealed distinct modifications in food preferences and eating patterns (Table 4). Overall, 34.2% of participants (*n* = 13) reported a shift in taste preferences. Specifically, 26.3% (*n* = 10) reported a distinct shift toward preferring savory foods over sweet foods, while 7.9% (*n* = 3) shifted toward sweet preferences, and 65.8% (*n* = 25) experienced no change in taste preference. Furthermore, a large majority of participants (84.2%, *n* = 32) reported a substantial reduction in overall meal portion sizes (score ≥7 on a 10-point Likert scale).

**Figure 1:**
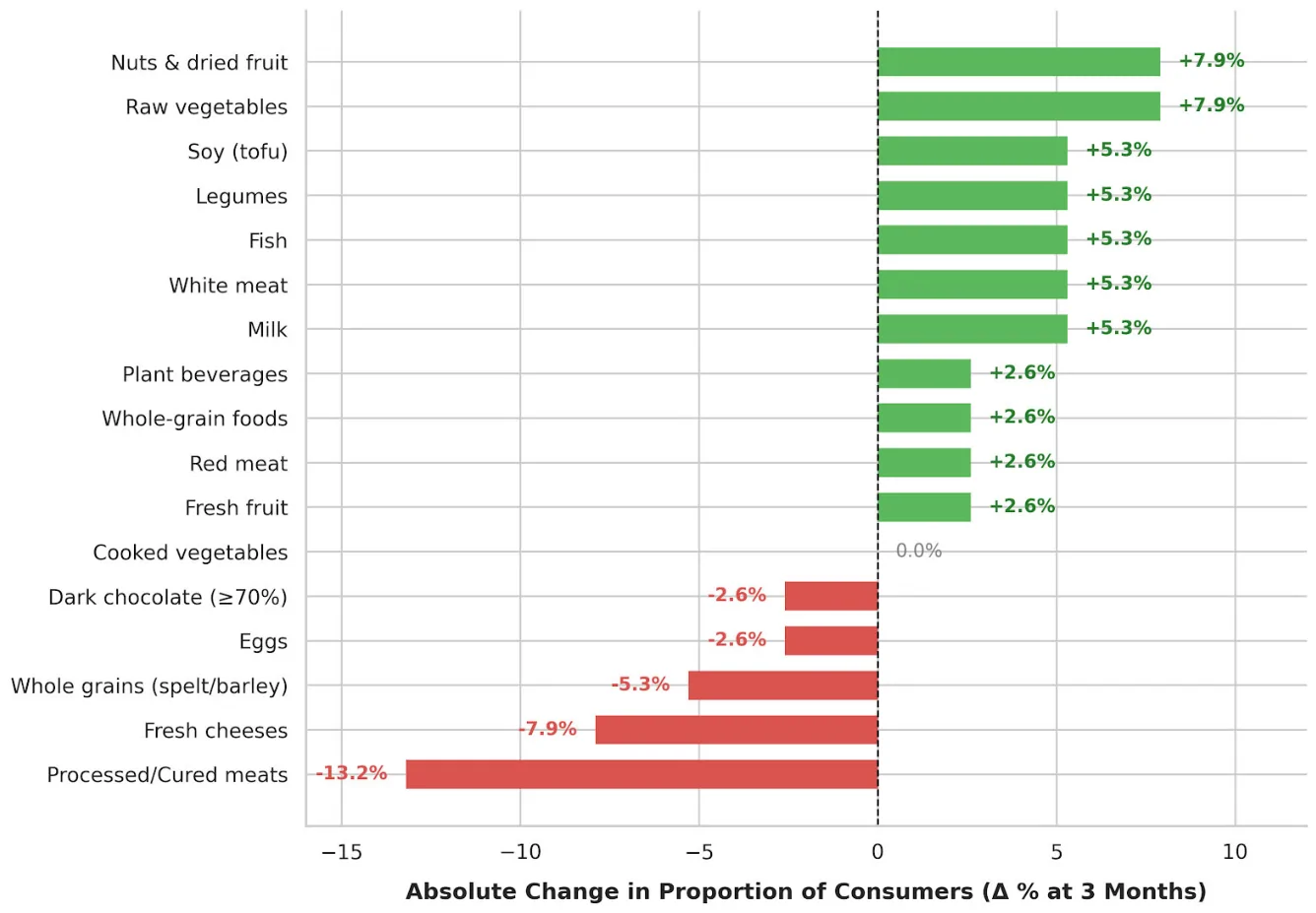
Absolute percentage changes in the proportion of regular consumers across specific food categories from baseline (T0) to 3 months (T1) (*N* = 38). Bars extending to the left (red) indicate a reduction in the proportion of participants consuming the specified food item, whereas bars extending to the right (green) reflect an increase. Data were derived from self-reported consumption (“YES” responses) on the structured dietary questionnaire.

Changes in the proportion of patients regularly consuming specific food groups at 3 months relative to baseline are illustrated in Figure 1. Changes in the reported frequency of consumption suggested qualitative shifts in food-group selection. However, these self-reported changes do not establish an improvement in overall dietary quality or nutritional adequacy. The most pronounced reductions in consumption frequency were observed for processed meats (−13.2%) and fresh cheeses (−7.9%). Conversely, the proportion of regular consumers increased for nutrient-dense and plant-based foods, including raw vegetables (+7.9%), nuts and dried fruits (+7.9%), legumes (+5.3%), fish (+5.3%), and white meat (+5.3%).

**Figure 2:**
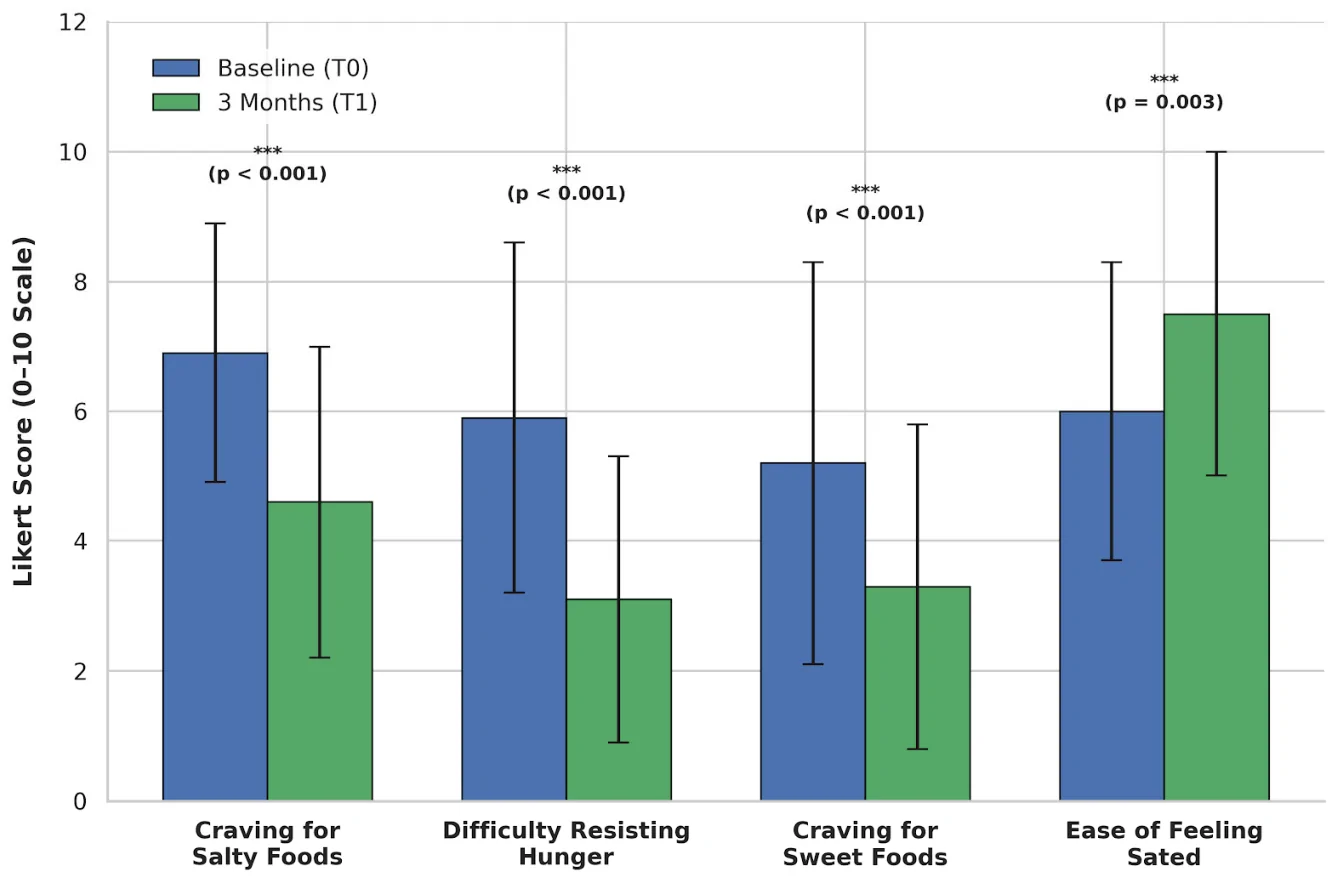
Mean scores (± SD) for appetite perceptions, food cravings, and meal satiety at baseline (T0) and 3 months (T1). Blue bars represent baseline values (T0) and green bars represent 3-month follow-up values (T1). All items were rated on a 0–10 Likert scale. Error bars denote standard deviation (SD). *** *p* < 0.005 derived from paired Student’s *t*-tests comparing T0 and T1.

Changes in appetite-related psychometric scores from baseline (T0) to 3-month follow-up (T1) are depicted in Figure 2. At the 3-month follow-up, statistically significant changes were observed in self-reported sweet and savory food cravings, difficulty resisting hunger between meals, and meal-induced satiety compared with baseline. Craving for savory foods dropped significantly from 6.9 ± 2.0 at baseline to 4.6 ± 2.4 at 3 months (*p* < 0.001), while craving for sweet foods declined from 5.2 ± 3.1 to 3.3 ± 2.5 (*p* < 0.001). Patients reported a substantial reduction in perceived difficulty in resisting hunger between meals (5.9 ± 2.7 vs. 3.1 ± 2.2, *p* < 0.001). Concurrently, the perceived ease of feeling sated during meals increased significantly from 6.0 ± 2.3 to 7.5 ± 2.5 (*p* = 0.003).

**Figure 3:**
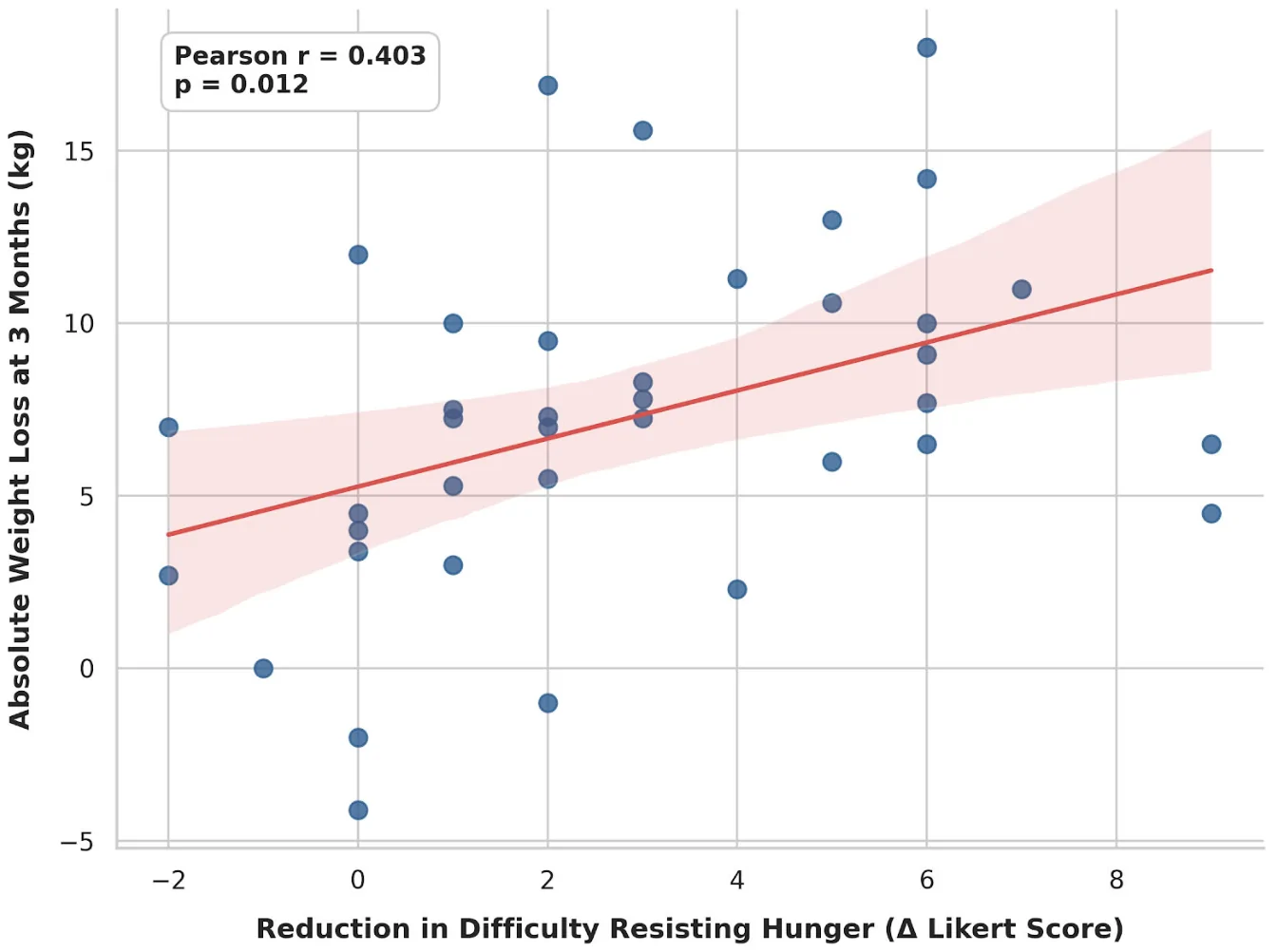
Correlation between reduction in difficulty resisting hunger between meals (Δ Likert score) and absolute weight loss (kg) at 3 months (*N* = 38). Each blue circle represents an individual patient. The solid red line depicts the linear regression trend, and the shaded band represents the 95% confidence interval. Statistical significance was assessed using Pearson’s correlation coefficient (*r* = 0.403, *p* = 0.012).

Correlation analyses were performed to explore associations between changes in self-reported appetite perceptions and absolute weight loss at 3 months (Figure 3). A statistically significant positive correlation was identified between the reduction in perceived difficulty in resisting hunger between meals (Δ Likert score) and total weight loss achieved at 3 months (*r* = 0.403, *p* = 0.012). Patients experiencing the greatest improvements in appetite control and hunger suppression achieved the most substantial reductions in body weight.

Gastrointestinal tolerability (nausea and perceived taste alterations) and changes in food-related pleasure were evaluated at 3 months (*N* = 38) (Table 5). Overall, participants reported a moderate mean score for nausea or taste alterations (4.8 ± 3.0 on a 10-point scale), with 55.3% (*n* = 21) reporting moderate-to-high symptoms (score ≥5). Symptoms were numerically higher in the Semaglutide group relative to Tirzepatide (5.5 ± 3.1 vs. 3.9 ± 2.5), though this difference did not reach statistical significance (*p* = 0.104). No statistically significant association was detected between nausea/taste alteration scores and 3-month weight loss (r = −0.148, *p* = 0.376). This finding does not exclude a contribution of these symptoms to weight change. Concurrently, patients experienced a moderate reduction in food-related pleasure (3.7 ± 1.9 out of 10), which was virtually identical between the Semaglutide (3.7 ± 2.1) and Tirzepatide (3.8 ± 1.6) cohorts (*p* = 0.871).

## 4. Discussion

Incretin-based therapies may influence body weight partly by changing how people experience and respond to food. In adults with obesity, short-term treatment with semaglutide has been associated with lower ad libitum energy intake, fewer food cravings, better control of eating, and a reduced relative preference for high-fat and energy-dense foods [13]. Similar findings have been reported with oral semaglutide in people with type 2 diabetes [14]. Emerging evidence suggests that tirzepatide also affects eating behavior. In a six-week randomized trial, tirzepatide reduced energy intake and altered brain responses to high-fat, high-sugar food cues [15]. Nevertheless, longer-term evidence remains limited. In the two-year STEP 5 trial, improvements in craving control and cravings for savory foods persisted with semaglutide, whereas some other measures showed less consistent differences at later assessments [16]. In another study, 20 weeks of semaglutide treatment resulted in greater fasting and postprandial appetite suppression than placebo. However, by weeks 40 and 60, the differences between the two groups were attenuated and no longer statistically significant [17]. Taken together, these findings suggest that the effects of incretin-based therapies on eating behavior may vary across behavioral domains and over time, supporting the need for further longitudinal studies.

In this prospective real-world study, three months of treatment with semaglutide or tirzepatide was accompanied by a significant reduction in body weight and blood pressure, lower self-reported sweet and savory food cravings, less perceived difficulty in resisting hunger between meals, greater meal-induced satiety, and a lower daily meal frequency. The observed changes are broadly consistent with previous studies showing that semaglutide reduces appetite and energy intake [13], and with secondary analyses of a phase 1 trial reporting reductions in food cravings and changes in food preferences during tirzepatide treatment [18]. The STEP 5 analysis also suggests that improvements in selected aspects of eating control can persist during longer-term semaglutide treatment [16]. Overall, our findings extend this literature by describing changes in self-reported appetite and eating behavior in patients treated in routine clinical practice.

Evidence for a role of central pathways comes from an experimental study in which the GLP-1 receptor agonist exenatide reduced responses to food images in brain regions involved in appetite and reward, including the insula, amygdala, and orbitofrontal cortex [19]. In our cohort, reductions in sweet and savory food cravings, together with lower perceived difficulty in resisting hunger between meals and greater meal-induced satiety, were consistent with changes in appetite-related perceptions during treatment. Correlation analysis showed that greater improvement in perceived hunger control was associated with greater weight loss, whereas nausea and taste alteration scores were not significantly associated with weight change. This observation is consistent with findings from studies in which gastrointestinal adverse events explained only a limited proportion of weight reduction during incretin-based therapy [20,21]. However, the absence of a statistically significant association in our sample does not exclude a contribution of gastrointestinal symptoms to individual treatment responses. Nor can the observed correlation establish that changes in appetite control mediated weight loss. Taken together, these findings support further investigation of the relationships between appetite regulation, food reward, gastrointestinal tolerability, and weight change during incretin-based treatment.

Beyond physiological hunger and satiety, eating behavior is influenced by food reward, responsiveness to environmental food cues, and the cognitive and motivational processes involved in food-related decisions. Emerging evidence suggests that semaglutide and tirzepatide may affect several of these domains, including food cravings, appetite, and the tendency to overeat. Real-world studies of semaglutide have reported reductions in self-reported cravings and emotional eating, while experimental studies of tirzepatide have described changes in appetite, food cravings, and responsiveness to food cues [15,22,23,24]. In our cohort, the reductions in sweet and savory food cravings and in perceived difficulty in resisting hunger between meals are consistent with changes in appetite-related perceptions during treatment. However, these findings should be interpreted within the specific behavioral domains assessed and do not establish changes in other cognitive or motivational aspects of eating behavior.

Furthermore, our study explored qualitative dietary changes accompanying incretin-based therapy. In a survey of 1955 US adults, Dilley and colleagues found that current and previous users of these medications reported reduced consumption, particularly of processed foods, sugar-sweetened beverages, refined grains, and beef, while fruit, leafy greens, and water were the only categories showing an overall increase. However, lower consumption was not consistently accompanied by lower food desirability, suggesting that reported intake and subjective food preferences may diverge during treatment [25]. In our study, 26.3% of participants reported a shift toward savory rather than sweet foods, while 84.2% reported a substantial reduction in meal portion size. The reported frequency of consumption also showed numerical changes in selected food categories, including lower consumption of processed meats and fresh cheeses and higher consumption of raw vegetables, nuts, legumes, fish, and white meat. However, these observations do not establish a statistically significant improvement in dietary quality. Daily meal frequency decreased significantly, whereas the reduction in snacking between main meals remained a nonsignificant trend. These findings highlight the importance of distinguishing changes in appetite, food preferences, portion size, and reported food-group consumption, as these outcomes represent related but different dimensions of eating behavior. Moreover, the marked reduction in portion size reported by most participants also has practical clinical implications. Reduced energy intake during GLP-1RA and dual GIP/GLP-1 receptor agonist therapy may compromise the intake of protein, fiber, fluids, and micronutrients, particularly when food choices are not guided, and may contribute to the loss of lean mass that accompanies weight reduction. Our findings support the integration of structured nutritional monitoring and counseling into pharmacological obesity care [26].

A further strength of this study is the direct comparison of semaglutide and tirzepatide in routine clinical practice. Patients receiving tirzepatide had a higher body weight and BMI at baseline than those receiving semaglutide. Over the three-month follow-up, the two groups showed numerically similar changes in body weight, appetite-related perceptions, and food cravings, and no statistically significant between-group differences were detected in the analyzed outcomes. These findings do not establish equivalent efficacy or exclude potentially relevant differences between treatments. Rather, they provide preliminary real-world data on the changes observed with both therapies and support further comparative studies designed and powered to assess differences in eating behavior and clinical response.

Several limitations should be acknowledged. First, the observational, non-randomized design and the absence of a control group prevent the attribution of the observed changes exclusively to pharmacological treatment. The influence of concomitant lifestyle modifications, study participation, and other unmeasured factors cannot be excluded. Second, the relatively small sample size limits statistical power, particularly for comparisons between semaglutide and tirzepatide, and reduces the generalizability of the findings. Third, the questionnaire was adapted from previously published tools but was not formally validated in the specific version used in this study. Its results should therefore be interpreted as exploratory self-reported measures and may be affected by recall and reporting bias. Moreover, the questionnaire did not provide an objective assessment of total energy intake or a comprehensive measure of dietary quality. Information on treatment indication, previous exposure to incretin-based therapies, and concomitant medications was obtained from clinical charts. Treatment adherence was monitored clinically during routine follow-up visits, but quantitative adherence measures were not systematically collected; therefore, differences in treatment adherence between participants could not be assessed. Biochemical parameters, including HbA1c and fasting plasma glucose, were not systematically available across the cohort and could not be analyzed.Finally, the three-month follow-up captures early changes only and cannot establish whether the observed behavioral and anthropometric changes persist over time. Further studies involving larger cohorts, longer follow-up, validated behavioral instruments, comprehensive clinical and metabolic assessments, and an appropriate control group are needed to confirm these preliminary findings.

## 5. Conclusions

Three months of treatment with semaglutide or tirzepatide was accompanied by weight loss and changes in self-reported eating behavior, including reduced sweet and savory cravings, improved perceived hunger control, and greater meal-induced satiety (Table 6). Participants also reported reductions in meal portion size and qualitative changes in food-group consumption. These findings provide preliminary evidence of behavioral changes occurring during treatment but do not establish that the observed changes were caused exclusively by the medications or mediated weight loss.

Summary of the primary qualitative, behavioral, and clinical insights derived from 3 months of incretin-based therapy in routine clinical practice.

## Figures and Tables

**Table 1:** Baseline Demographic, Anthropometric, Clinical, and Behavioral Characteristics of the Study Population (*N* = 38).

Characteristic at Baseline (T0)	Total Cohort (N = 38)	Male (n = 10)	Female (n = 28)	Semaglutide (n = 21)	Tirzepatide (n = 17)	*p*-Value (Gender) †	*p*-Value (Drug) ‡
Demographic Measures							
Age—yr	56.1 ± 11.3	52.8 ± 11.8	57.2 ± 11.1	56.3 ± 9.4	55.7 ± 13.5	0.318	0.873
Anthropometric & Hemodynamic Measures							
Height—cm	166.7 ± 10.4	179.9 ± 4.5	162.0 ± 7.4	165.4 ± 10.1	168.4 ± 10.9	<0.001	0.380
Weight—kg	91.1 ± 14.5	102.6 ± 12.7	87.0 ± 13.0	83.9 ± 11.1	100.1 ± 13.3	0.004	<0.001
Body-mass index—kg/m^2^	32.7 ± 4.5	31.7 ± 3.2	33.0 ± 4.8	30.5 ± 3.4	35.4 ± 4.2	0.323	<0.001
Systolic blood pressure—mm Hg	137.4 ± 9.2	139.5 ± 8.5	136.6 ± 9.5	134.9 ± 9.1	140.5 ± 8.7	0.390	0.060
Diastolic blood pressure—mm Hg	85.6 ± 5.3	87.1 ± 8.6	85.1 ± 3.5	86.3 ± 4.9	84.8 ± 5.7	0.484	0.409
Dietary Habits & Appetite Perception							
Daily meal frequency—meals/day	4.3 ± 1.3	4.3 ± 1.1	4.3 ± 1.4	4.2 ± 1.4	4.4 ± 1.3	0.974	0.617
Daily fluid intake—L/day	1.4 ± 0.7	1.5 ± 0.5	1.4 ± 0.7	1.6 ± 0.7	1.2 ± 0.7	0.627	0.133
Craving for sweet foods (0–10 scale)	5.2 ± 3.1	4.1 ± 3.0	5.6 ± 3.1	4.8 ± 3.1	5.7 ± 3.1	0.202	0.362
Craving for savory foods (0–10 scale)	6.9 ± 2.0	7.1 ± 1.7	6.9 ± 2.1	7.0 ± 2.1	6.9 ± 1.9	0.722	0.914

Baseline characteristics are summarized as mean ± standard deviation (SD). The study cohort comprised 38 patients at baseline (T0) initiating treatment with a GLP-1 receptor agonist or a dual GIP/GLP-1 receptor agonist. Between-group continuous baseline comparisons were performed using Welch’s two-sample *t*-test, whereas categorical proportions were compared using Fisher’s exact test. † *p*-value refers to baseline comparison between male (*n* = 10) and female (*n* = 28) subgroups. ‡ *p*-value refers to baseline comparison between Semaglutide (*n* = 21) and Tirzepatide (*n* = 17) treatment arms. BMI indicates body-mass index; BP, blood pressure.

**Table 2:** Changes in Anthropometric, Hemodynamic, Behavioral, and Appetite Perception Parameters After 3 Months of Incretin-Based Therapy.

Parameter	Baseline (T0)	3 Months (T1)	Absolute Change (Δ T1–T0)	*p*-Value †
Anthropometric and Hemodynamic Parameters				
Body Weight—kg	91.1 ± 14.5	83.9 ± 14.9	−7.2 ± 4.9	<0.001
Body-mass index (BMI)—kg/m^2^	32.7 ± 4.5	30.0 ± 4.9	−2.6 ± 1.6	<0.001
Systolic blood pressure (SBP)—mm Hg	137.4 ± 9.2	131.3 ± 10.3	−6.1 ± 6.3	<0.001
Diastolic blood pressure (DBP)—mm Hg	85.6 ± 5.3	83.6 ± 6.7	−2.0 ± 5.9	0.042
Dietary Habits and Behavioral Measures				
Daily meal frequency—meals/day	4.3 ± 1.3	3.6 ± 1.0	−0.7 ± 1.4	0.005
Daily fluid intake—L/day	1.4 ± 0.7	1.6 ± 0.7	+0.1 ± 0.6	0.201
Appetite Perception and Food Cravings				
Craving for sweet foods (0–10 scale)	5.2 ± 3.1	3.3 ± 2.5	−1.9 ± 2.7	<0.001
Craving for savory foods (0–10 scale)	6.9 ± 2.0	4.6 ± 2.4	−2.3 ± 2.7	<0.001
Difficulty resisting hunger between meals (0–10 scale)	5.9 ± 2.7	3.1 ± 2.2	−2.8 ± 2.9	<0.001
Ease of feeling sated during a meal (0–10 scale)	6.0 ± 2.3	7.5 ± 2.5	1.5 ± 2.9	0.003

Plus–minus values are means ± standard deviation (SD). All measurements were obtained from the prospective paired cohort of participants completing both baseline (T0) and 3-month follow-up (T1) evaluations (*N* = 38). † *p*-values were calculated using two-tailed paired Student’s *t*-tests comparing T0 and T1 values. Abbreviations: BMI, body-mass index; DBP, diastolic blood pressure; SBP, systolic blood pressure; T0, baseline evaluation prior to therapy initiation; T1, 3-month follow-up evaluation.

**Table 3:** Comparison of 3-Month Mean Changes (Δ T1–T0) in Clinical, Hemodynamic, and Behavioral Parameters Between Semaglutide and Tirzepatide Treatment Arms.

Parameter Change (Δ T1–T0)	Semaglutide (*n* = 21)	Tirzepatide (*n* = 17)	*p*-Value †
Anthropometric and Hemodynamic Changes			
Body Weight—kg	−7.2 ± 4.5	−7.3 ± 5.5	0.924
Body-mass index (BMI)—kg/m^2^	−2.4 ± 1.4	−3.0 ± 1.9	0.258
Systolic blood pressure (SBP)—mm Hg	−6.6 ± 5.9	−5.5 ± 6.9	0.589
Diastolic blood pressure (DBP)—mm Hg	−3.0 ± 4.8	−0.8 ± 7.0	0.278
Dietary Habits and Behavioral Changes			
Daily meal frequency—meals/day	−0.5 ± 1.5	−0.8 ± 1.2	0.499
Daily fluid intake—L/day	0.1 ± 0.6	0.2 ± 0.6	0.696
Appetite Perception and Craving Changes			
Craving for sweet foods (0–10 scale)	−1.5 ± 2.6	−2.2 ± 2.9	0.435
Craving for savory foods (0–10 scale)	−2.7 ± 2.7	−1.8 ± 2.6	0.281
Difficulty resisting hunger between meals (0–10 scale)	−2.8 ± 3.0	−2.9 ± 2.8	0.849
Ease of feeling sated during a meal (0–10 scale)	1.1 ± 2.7	2.1 ± 3.1	0.324

Plus–minus values represent mean absolute change ± standard deviation (Δ=*T*1 − *T*0) from baseline (T0) to 3-month follow-up (T1) † *p*-values were calculated using Welch’s two-sample *t*-test comparing the mean changes (Δ) between the Semaglutide (*n* = 21) and Tirzepatide (*n* = 17) treatment subgroups. Abbreviations: BMI, body-mass index; DBP, diastolic blood pressure; SBP, systolic blood pressure; T0, baseline evaluation; T1, 3-month follow-up evaluation.

**Table 4:** Qualitative Changes in Food Preferences, Taste Perception, and Meal Portion Size at 3 Months.

Outcome Domain	Patients (N = 38)	Percentage (%)
Shift in Taste Preferences (Sweet vs. savory)		
—No change in preference	25	65.8
—Shift toward savory/savory foods	10	26.3
—Shift toward sweet foods	3	7.9
Meal Portion Size Reduction		
—Substantial portion reduction (Likert Score ≥ 7/10)	32	84.2
—Moderate/minor portion reduction (Likert Score < 7/10)	6	15.8

Data are presented as counts (*n*) and relative percentages (%). All data were derived from the self-administered, structured questionnaire at 3-month follow-up (T1). Substantial meal portion reduction was defined as a score ≥7 on a 10-point Likert scale.

**Table 5:** Gastrointestinal Symptoms, Taste Alterations, and Food Hedonic Scores at 3-Month Follow-Up Stratified by Incretin Therapy.

Outcome Measure at 3 Months (T1)	Total Cohort (N = 38)	Semaglutide (n = 21)	Tirzepatide (n = 17)	p-Value †
Gastrointestinal & Taste Perception				
—Nausea or taste alteration score (0–10 scale)	4.8 ± 3.0	5.5 ± 3.1	3.9 ± 2.5	0.104
—Moderate-to-high symptoms (% with score ≥ 5)	21 (55.3%)	13 (61.9%)	8 (47.1%)	0.362
Food Hedonics				
—Reduction in food-related pleasure (0–10 scale)	3.7 ± 1.9	3.7 ± 2.1	3.8 ± 1.6	0.871

Plus–minus values are means ± standard deviation (SD). All ratings were self-reported on a 0–10 Likert scale at the 3-month follow-up evaluation (*T*1). † *p*-values were calculated using Welch’s two-sample *t*-test for continuous variables and Fisher’s exact test for categorical proportions comparing the Semaglutide (*n* = 21) and Tirzepatide (*n* = 17) treatment groups.

**Table 6:** Key Take-Home Messages: Behavioral and Clinical Impact of Incretin Therapies.

Domain/Core Finding	Key Clinical Take-Home Message
Central Mechanism of Action	Weight loss was associated with improvements in self-reported hunger control; the underlying mechanisms remain uncertain.
Craving & Satiety Reshaping	Incretin therapy was accompanied by reduced self-reported cravings for sweet and savory foods and greater meal-induced satiety. A reduction in snacking between main meals was observed as a non-significant trend.
Spontaneous Dietary Improvement	Changes in reported food-group consumption were observed, but an improvement in overall dietary quality was not established.
Comparable Drug Efficacy	No statistically significant differences in the analyzed changes were detected between treatment groups; the study was not designed to establish equivalence.

## Data Availability

The datasets produced and analysed during the present study are obtainable from the corresponding author upon reasonable request.

## Supplementary Materials

The following supporting information can be downloaded at: https://www.mdpi.com/article/10.3390/nu18213675/s1, Figure S1: Participant flow diagram. Flow of participants through enrolment, initiation of incretin-based therapy, follow-up, and final analysis.

## Abbreviations

Body mass index (BMI), blood pressure (BP), diastolic blood pressure (DBP), glucose-dependent insulinotropic polypeptide (GIP), glucagon-like peptide-1 (GLP-1), glucagon-like peptide-1 receptor agonist (GLP-1RA), systolic blood pressure (SBP), standard deviation (SD), baseline evaluation (T0), 3-month follow-up evaluation (T1).
