# Supplementary material for: Changes in Eating Behavior and Food Preferences After Semaglutide or Tirzepatide Initiation: A Prospective Observational Pilot Study
Source: Nutrients. 2026 Nov 5;18(21):3675. doi: 10.3390/nu18213675 (PMC13765408; doi:10.3390/nu18213675)
Supplement: Supplementary file 1 [file nutrients-18-03675-s001.zip › nutrients-4643624-supplementary.pdf]

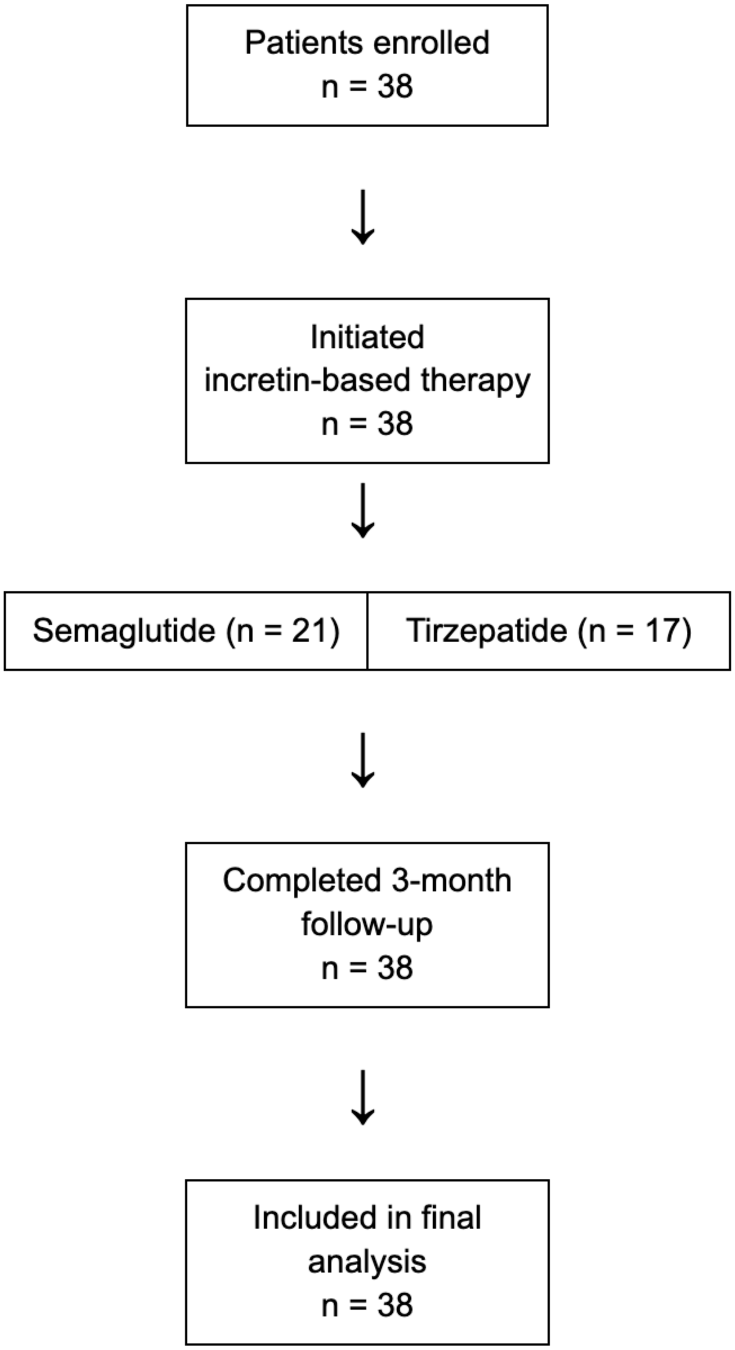

Figure S1. Participant flow diagram. Flow of participants through enrolment, initiation of incretin-based therapy, follow-up, and final analysis.
